# Emergence and Full Genome Analysis of *Tomato Torrado Virus* in South Africa

**DOI:** 10.3390/v12101167

**Published:** 2020-10-15

**Authors:** Vaneson Moodley, Augustine Gubba, Paramu L. Mafongoya

**Affiliations:** 1 Discipline of Plant Pathology, School of Agricultural, Earth and Environmental Sciences, University of KwaZulu-Natal, Scottsville, Pietermaritzburg 3209, South Africa; GubbaA@ukzn.ac.za; 2 Rural Agronomy and Development, School of Agricultural, Earth and Environmental Sciences, University of KwaZulu-Natal, Scottsville, Pietermaritzburg 3209, South Africa; mafongoya@ukzn.ac.za

**Keywords:** ToTV, emerging disease, prevalence, whole-genome sequencing, phylogeny

## Abstract

Emerging pests and diseases are a major threat to food production worldwide. In a recent survey, *Tomato torrado virus* (ToTV) was identified on tomato crops in the Limpopo province of South Africa and a first report of the disease was published. In this follow-up study, the full genome sequence of a tomato-infecting isolate of ToTV from South Africa was elucidated. High-throughput sequencing was used to generate the full genome of ToTV infecting tomato crops in South Africa. The longest contig obtained for the RNA-1 and RNA-2 genome of ToTV was comprised of 7420 and 5381 nucleotides (nt), respectively. Blast analysis of the RNA-1 sequence of ToTV from South Africa (ToT-186) matched 99% to a Spanish and Polish isolate; the RNA-2 segment of ToTV from South Africa (ToT-186) matched 99% to ToTV isolates from Italy and Poland, respectively. The information presented in this study will go a long way towards better understanding the emergence and spread of ToTV and devising sustainable management of ToTV diseases.

## 1. Introduction

Emerging pests and disease have destroyed agricultural crops all over the world. More than fifteen years ago, local farmers in the city of Murcia (located in the southeastern parts of Spain) observed severe necrosis on the leaves and fruit of tomato crops. These symptoms later became known as “torrado” disease which was coined by local Spanish farmers describing the “burnt-like” effect of the disease in tomato fields. A subsequent study by Verbeek et al. [1] provided a detailed analysis of a new picorna-like virus infecting tomatoes which they termed “*Tomato torrado virus*”. Although this newly discovered species was shown to display several characteristics similar to the Sequivirus, Sadwavirus, and Cheravirus genera in the Sequiviridae family, sequence characteristics distinguished *Tomato torrado virus* (ToTV) as a member of a new plant virus genus [1].

According to Sanfacon et al. [2]. ToTV is the type member of the genus Torradovirus in the family Secoviridae which is an amalgamation of the families Sequiviridae and Comoviridae, together with previously unassigned genera Cheravirus and Sadwavirus, in the order Picornavirales. In addition, members of the Secoviridae family have a small icosahedral particle morphology (25–30 nm) with a pseudo-T = 3 symmetry, and a mono/bipartite positive-strand RNA genome. The capsid of non-enveloped virions contain jelly-roll domains that are organized into three mature capsid proteins that are folded alike but vary in amino acid sequence and length [3]. These domains may have resulted from the triplication of a single domain and consecutive divergent evolution [4].

Torradoviruses have a bipartite genome composed of an RNA-1 (7.8 kb) and an RNA-2 (5.4 kb) segment. Each genomic segment has a VPg linked to its 5′ end and a 3′ poly (A) tract. RNA-1 and RNA-2 are translated into two polyproteins, which are then processed into functional proteins. RNA-1 encodes proteins (a type III helicase, 3C-like proteinase, and a type I RNA-dependent RNA-polymerase) that are necessary for replication while RNA-2 encodes three coat proteins and proteins involved in virus movement. It is further demonstrated that the genome of torradoviruses consists of an exclusive second open reading frame (ORF) upstream of RNA-2 which partially overlaps the large ORF and encodes a protein of unknown function that displays a great degree of sequence diversity with other torradoviruses [2]. The large 3′ NTR shares > 99% sequence identity between the RNA-1 and RNA-2 segments of a particular torradovirus species but varies substantially in terms of interspecific differences.

ToTV along with three other torradovirus species i.e., *Tomato marchitez virus* (ToMarV), *Tomato chocolàte virus* (ToChV), and *Tomato necrotic dwarf virus* (ToNDV) are presently the only known spherical viruses that are transmitted by three whitefly species, i.e., *Trialeurodes vaporariorum*, *Bemisia tabaci*, and *Trialeurodes abutilonea* in a semi-persistent manner [5,6]. Verbeek et al. [5] further demonstrate that the virus is retained in the stylet where it may remain for up to eight hours without loss of transmission efficiency. The presence of whitefly species has been identified to varying degrees throughout the world. Studies of emerging whitefly-transmitted viruses such as ToTV have been linked to abnormally high vector populations [7]. Jones et al. [8] indicate that *B*. *tabaci* and *T*. *vaporariorum* thrive in warmer temperatures with prolonged dry spells of up to four months (<80 mm rainfall/month), although *T*. *vaporariorum* is more tolerant of cold weather.

South Africa recently experienced a severe drought which was exacerbated by the effects of *El Niño*. Provinces situated in the north experienced extremely high temperatures and prolonged periods of dry weather that resulted in millions of dollars in crop losses. During a national survey, an unprecedented whitefly infestation was observed in major tomato growing areas throughout South Africa. Tomato growers in the Limpopo province (the largest producer of tomatoes in South Africa) noticed severe necrosis/burnt-like symptoms on the leaves, stems, and fruit that resembled heat burn or possibly osmotic stress that usually results from an excess of nitrogen salts (personal communication). Closer observation revealed that the “burnt like” symptoms were similar to those described by the tomato farmers in Murcia. Primary symptoms appeared as necrotic spots surrounded by chlorotic halos that began at the base of immature leaves, and advanced as stunted growth, vertical stem necrosis, and necrotic spots on the fruit resulting in significant yield losses in affected areas.

RT-PCR assays showed that ToTV was only present on tomato crops and *Datura stramonium* (jimson weed) samples that were collected from the Limpopo province (situated far north of South Africa). In addition, an arable weed species (*Abutilon grantii* Meeuse; appearing on SANBI’s (South African National Biodiversity Institute) red list) (http://redlist.sanbi.org/species.php?species=2595-13) growing among tomato crops in the northern part of the KwaZulu Natal province tested positive for ToTV infection (TorKZN-186, KY581570). No other torradovirus species, including *Tomato marchitez virus* (ToMarV), *Tomato chocolàte virus* (ToChV), *Tomato necrotic dwarf virus* (ToNDV), and *Tomato chocolate spot virus* (ToChSV), were identified in South Africa. Additionally, all whitefly-infested symptomatic bell pepper plants that were assayed for the presence of torradovirus infections using RT-PCR were negative.

A total of 316 tomato samples, 269 bell pepper samples, and 182 weed samples belonging to six botanical families (Amaranthaceae, Asteraceae, Brassicaceae, Euphorbiaceae, Malvaceae, and Solanaceae) were tested for torradovirus infection. A high prevalence of ToTV was identified on tomato (71.6%) and D. stramonium (66.7%) in the Limpopo province of South Africa [9]. To a lesser extent, ToTV was identified on the arable weed (*Abutilon grantii*) growing among tomato crops in the northern KwaZulu Natal province (13.8%); adjacent/nearby tomato crops were not infected [9]. ToTV infection of tomatoes was restricted to the Limpopo province which also had comparably higher whitefly infestation levels. Overall, only 15.1% of tomatoes and 11% of weeds sampled from South Africa tested positive for ToTV infection [9].

Globally, ToTV has been identified in Spain, Hungary, Poland, Canary Islands, France, Panama, Italy, Australia, Colombia, and Morocco [1,10,11,12,13,14,15,16,17,18]. During a national survey in 2015, ToTV was identified for the first time in South Africa [9,19]. In this follow-up study, we report the first full genome sequence and phylogenetic analysis of a South African isolate of ToTV from tomato.

## 2. Materials and Methods

### 2.1. RNA Extraction

Total RNA was extracted using a Quick-RNA™ MiniPrep kit (Zymo Research, Waltham, MA, USA) according to the manufacturers’ guidelines. Six hundred microliters of lysis buffer was added to 20 mg of frozen leaf tissue in a sterile 1.5 mL microcentrifuge tube containing five to six tanzanite beads. Samples were subsequently macerated using a bead beater homogenizer. Prior to the final elution step, 30 µL of nuclease-free water was added to the column and allowed to incubate for 2 min at room temperature. The quality and quantity of each RNA extract was measured using a Nanodrop 2000 spectrophotometer (Thermo Fisher Scientific Inc., Waltham, MA, USA). Samples were stored at −80 °C pending further analysis.

### 2.2. Reverse Transcription–Polymerase Chain Reaction (RT-PCR)

A two-step RT-PCR approach was used as the first measure to detect torradovirus infections due to the lack of commercially available antibodies. Template RNA (4 µL) was incubated at 65 °C for 5 min and subsequently kept on ice. A master mix component containing 2 µL of a gene-specific primer, 4 µL of reaction buffer, 1 µL of reverse transcriptase enzyme, 1 µL of ribolock RNase inhibitor, 2 µL of dNTPs, and 6 µL of nuclease-free water was added to make a final volume of 20 µL for each reaction. cDNA was synthesized using a RevertAid Premium Reverse Transcriptase kit (Thermo Fisher Scientific Inc., Waltham, MA, USA) according to the manufacturers’ guidelines. Conditions for RT were 42 °C for 1 h and 70 °C for 10 min.

PCR was carried out in 20 µL reaction volumes using a KAPA2G Fast HotStart ReadyMix PCR kit (KAPA Biosystems, Wilmington, NC, USA). Each PCR reaction contained 10 µL of KAPA Ready Mix, 2 µL of each primer (10 ng/µL), 30 ng of template DNA, and nuclease-free water. A set of degenerate torradovirus primers Torrado-2F (corresponding to nt 2589–2608 of ToTV (PRI-ToTV0301; DQ388880); nt 2528–2547 of ToMarV (PRI-TMarV0601; EF681765); nt 2561–2580 of ToChSV (GQ305132) and nt 2568–2587 of ToChV (ToChV-G01; FJ460290)) and Torrado-2R (corresponding to nt 3084–3103 of ToTV (PRI-ToTV0301; DQ388880); nt 3023–3042 of ToMarV (PRI-TMarV0601; EF681765); nt 3056–3075 of ToChSV (GQ305132) and nt 3063–3082 of ToChV (ToChV-G01; FJ460290)) that target a 515 bp region (overlapping the Vp35 and Vp26) located on the RNA-2 strand was used to detect the presence of torradoviruses [17]. Conditions for PCR were 95 °C for 2 min; 35 cycles of 95 °C for 30 s, 51 °C for 25 s and 72 °C for 20 s followed by a final elongation at 72 °C for 10 min. PCR products were resolved on a 1.5% agarose gel stained with SYBR Safe DNA gel stain (Invitrogen, Carlsbad, CA, USA).

### 2.3. Cloning and Sequencing

PCR-positive amplicons were excised and purified using a Zymoclean Gel DNA Recovery Kit (Zymo Research, Irvine, CA, USA). A TA cloning kit (Invitrogen, Carlsbad, CA, USA) was used to ligate the target sequences from purified gel extracts onto a PCR 2.1 cloning vector following the manufacturers’ guidelines. Chemically competent *Escherichia coli* cells were transformed by heat shock (42 °C for 30 s) prior to a 30 min incubation on ice. Successful transformants were selected using blue/white colony screening and cultured overnight at 37 °C in Luria–Bertani (LB) broth containing 50 µg/mL kanamycin. Plasmid extractions were carried out using a Zyppy Plasmid Miniprep Kit (Zymo Research, Irvine, CA, USA). The insert was confirmed using *Eco*R1 endonuclease activity (Thermo Fisher Scientific, Waltham, MA, USA). Reactions were incubated at 37 °C for 15 min and terminated at 85 °C for 5 min. Bi-directional sequencing of positive transformants were carried out at Inqaba Biotec (Pretoria, South Africa) using a 3500xL Genetic Analyzer (Applied Biosystems, Foster City, CA, USA). The Blast tool in MEGA X software [20] was used to validate the identity of each clone against sequences available on the NCBI GenBank database.

### 2.4. Genome Analysis

Total RNA was extracted (>50 ng/uL) from a ToTV-positive tomato (*Solanum lycopersicum* Mill.) plant collected from the Limpopo province (DMS: 23°38′22.6104″ S 30°4′40.2996″ E) and analyzed using high-throughput sequencing (HTS). HTS data were generated using an Illumina HiSeq 2500 Ultra-High-Throughput Sequencing System (Illumina Inc., Santiago, CA, USA) at the Agricultural Research Council Biotechnology Platform (ARC-BTP (Pretoria, South Africa)), and the raw data were deposited into GenBank SRA: SUB8159355. Read lengths less than 25 nucleotides were trimmed and pair-end sequence libraries were generated. The raw data were trimmed using Trimmomatics version 0.36 whereby the low-quality sequence regions and Illumina universal adapter sequences were trimmed and removed. CLC genomics workbench 9.5.3 (https://www.qiagenbioinformatics.com/) was used to remove host data by matching sequence reads to a reference tomato genome (Heinz, Accession no. NC015449) prior to de novo assembly. The remaining contigs were then identified using Blast version 2.6.0. against the NCBI nucleotide database. Contig sequences with viral identities were extracted for functional annotation with Blast2GO using the default parameters. Protein translation and ORFs were identified using ORF Finder (https://www.ncbi.nlm.nih.gov/orffinder/). A comparison of nucleotide and amino acid similarities was established using SIAS tools (http://imed.med.ucm.es/Tools/sias.html), and phylogenetic analysis of the RNA-1 and RNA-2 genome of ToTV was inferred from trees generated using a best-fit model in MEGA 6 software. The sequence of each RNA segment generated from the HTS analysis was reconstructed and verified with primers designed using SnapGene v5.1.4.1 (Tables S1 and S2). The genome of ToTV infecting tomato crops in South Africa was constructed from overlapping RT-PCR clones.

## 3. Results

### 3.1. Survey Analysis

**Figure 1:**
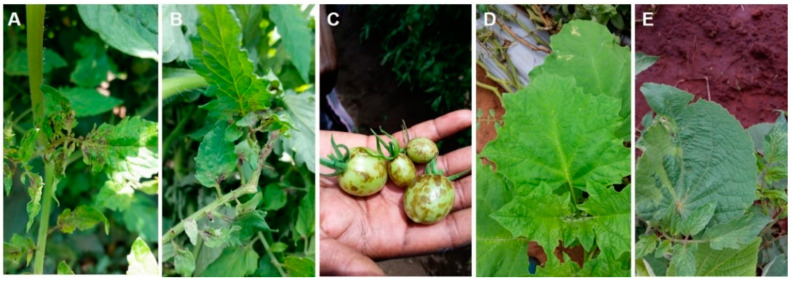
Tomato crops and nearby weed species exhibiting virus-like symptoms. (**A**): Necrotic spots beginning at the base of young leaves. (**B**): Vertical stem necrosis. (**C**): Necrotic spots and fruit deformation. (**D**): Chlorosis, necrotic spots, stunted growth, and linear chlorotic spots along the veins of *Datura stramonium*. (**E**): Stunting, chlorosis, and leaf deformation symptoms on the arable weed *Abutilon grantii* growing among tomato field crops in the northern KwaZulu Natal province.

The emergence of torradovirus-like symptoms in the Limpopo province of South Africa reduced the yield and quality of tomatoes. In severely affected crops, fruit set was suppressed (Figure 1A–C). ToTV symptoms on tomato fruit (Figure 1C) were often mistaken for *Tomato spotted wilt virus* (TSWV) by local tomato growers and government extension workers. Nearby weeds infested with whiteflies exhibited symptoms such as stunting and leaf deformation. (Figure 1D).

Many of the tomato crops exhibiting torradovirus-like symptoms were concentrated in the northern parts of South Africa (particularly in the Limpopo province). Based on phenotypic analysis, both *Trialeurodes* sp. and *Bemisia* sp. were present on symptomatic field and greenhouse cultivated tomato crops. Symptom severity was heightened in areas located in the northern parts of South Africa where whitefly populations were significantly higher.

### 3.2. Virus Detection

Each 515 bp PCR positive product was validated by cloning and sequencing. A consensus sequence was derived from the isolates of ToTV infecting tomato crops and *D*. *stramonium* in the Limpopo province of South Africa based on their nucleotide sequence similarity. Blast analysis showed that the isolate of ToTV from South Africa (Lim-186, KP890356) matched 99% to the Polish isolate Walʹ03 (EU563947) [19,21]. The isolate of ToTV identified on *Abutilon grantii* (family Malvaceae) growing among tomatoes in the northern KwaZulu Natal province (TorKZN-186, KY581570) was not identified on tomato crops and matched 92.8% to the isolate T795 (KX132809) from Italy [9].

### 3.3. Sequence Analysis

The full genome of ToTV was elucidated using high-throughput sequencing. The dataset contained 23,624,259 raw paired-end reads. A total of 127,654 contigs were generated from the de novo assembly. Only 2101 contigs did not align with the host genome and consisted of viral, bacterial, fungal, and traces of plant sequences. Of the 2101 contigs, 68 contigs were similar to known viral sequences. The longest contig obtained for the RNA-1 and RNA-2 genome of ToTV was comprised of 7420 and 5381 nucleotides (nt), respectively. Sequences generated from each RT-PCR clone (amplified with the primers listed in Tables S1 and S2) that were obtained from two individual plant extracts matched to designated regions on the RNA-1 and RNA-2 segment of ToT-186, respectively, confirming the absence of quasispecies.

### 3.4. RNA-1

**Figure 2:**
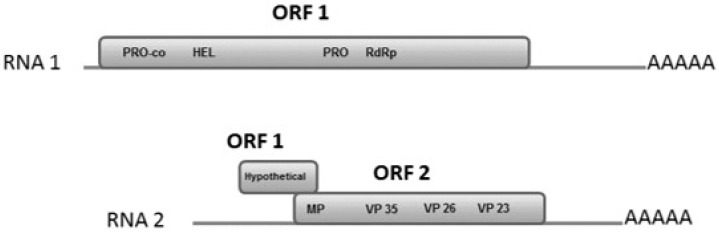
Bipartite genome organization of torradoviruses. RNA-1 encodes a large polyprotein (ORF-1) that contains a protease cofactor (PRO-co), helicase (HEL), 3C-like protease (PRO), and an RNA-dependent RNA polymerase (RdRp) which are involved in proteolytic cleavage and replication. RNA-2 encodes a hypothetical protein that partially overlaps ORF 2. Motifs associated with a movement protein (MP) and three coat proteins are present in ORF-2.

RNA-1 (ToT-186; Accession no. MH587229) was 7420 nt in length excluding the poly (A) tail and comprised of a 109 nt 5′ untranslated region, a single open reading frame (ORF-1), and an 834 nt 3′ non-coding region (Figure 2). ORF-1 (nucleotides 110–6586) encodes a predicted 241 kDa polyprotein (6477 nt; 2158 amino acids (aa)) and contains an initiation codon (AUG), and stop codon (UGA) at positions 110–112 nt and 6584–6586 nt, respectively. According to Verbeek et al. [1], there are conserved regions in the polyprotein with motifs typically associated with a protease cofactor (PRO-co), helicase (HEL), protease (PRO) and an RNA dependent RNA polymerase (RdRP) (Figure 2). Their functional domains are PRO-co (aa 106–338), HEL (aa 337–534), 3C-like PRO (aa 1000–1100), RdRP (aa 1303–1554). The position of each domain was determined by high nucleotide (>98%) and amino acid (>99%) sequence similarities with Polish (KJ940975) and Spanish (DQ388879) isolates Table 1. Consistently higher nucleotide and amino acid sequence similarity patterns were observed among members of each torradovirus species. Interestingly, the nucleotide sequence analysis of the 3ʹ non-translated region showed a high level of inter and intra-specific variation among isolates. Blast analysis of the RNA-1 sequence of ToTV from South Africa (ToT-186) matched 99% to the Spanish isolate (DQ388879) and Polish isolate Kra (KJ940975).

### 3.5. RNA-2

RNA-2 (ToT-186; Accession no. MH587230) spanned a total of 5381 nucleotides (nt) excluding the polyadenylated tail and comprised of two ORFs, a 172 nt 5′ leader sequence and a large non-coding region (1092 nt) at the 3′ end (Figure 2). ORF-1 (nucleotides 173–736) encodes a 20 kDa protein (564 nt; 187 aa) with no known function or homology to other proteins in the database. ORF-2 (nucleotides 693–4289) partially overlaps ORF-1 and encodes a large 133.5 kDa polyprotein (3597 nt; 1198 aa) which includes three virion capsid subunits [21]. The coat proteins Vp35 (amino acids 483–728), Vp26 (amino acids 733–969), Vp23 (amino acids 982–1198) have a molecular weight of approximately 35 kDa, 26 kDa, and 23 kDa, respectively. A movement protein consensus sequence (LxxPxL) identified near the N-terminal region of ORF-2 indicates the likelihood of a putative movement protein [22]. Budziszewska et al. [21] established that the putative 3A movement protein (MP) encoded by ORF-2 near the N-terminal is common to both ToTV and ToMarV. Alignment of the MP indicates that it is encoded in a similar position as all tomato-infecting torradovirus species. Nucleotide and amino acid similarities of the 3A protein (Table 2) show that all ToTV species share a very high (>98% nt) and (100% aa) similarity with each other but vary by approximately 70% (nt) and 80% (aa) when compared with other torradovirus species. Interestingly, all other torradovirus species, including ToMarV, ToChV, ToChSV, and ToNDV, share equally high similarities with each other. High levels of variability are seen in the 5′ leader sequence among different torradovirus species. ToMarV and ToNDV had the highest level of nucleotide variability in the 5′ UTR (35–37% nt) when compared to ToTV isolates. All torradoviruses characteristically have a short 5′ leader sequence and an unusually long 3′ non-coding region (NCR). The 3′ NCR of ToTV extends approximately 1098 nt and ToChV more than 1400 nt. Additionally, analysis of the 3′ NCR showed that ToChV shared the lowest nucleotide similarity (<32%) with ToTV isolates. Although the percentage of nucleotide and amino acid similarities in the coat protein (CP) are conserved among torradovirus species, the VP35 showed higher levels of variability between ToTV isolates and other torradovirus species when compared to the VP26 and VP23. Blast analysis of the RNA-2 segment of ToTV from South Africa (ToT-186) matched 99% to ToTV isolates T795 (KX132809) and Ros (KM114266) from Italy and Poland, respectively.

### 3.6. Phylogeny

**Figure 3:**
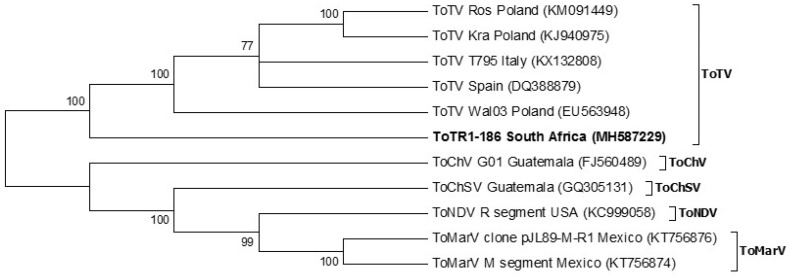
Phylogenetic relationship of the full-length ToTV RNA-1 genome from South Africa (ToTR1-186) with all other fully sequenced tomato-infecting torradovirus isolates to date. Evolutionary analysis was inferred using the maximum likelihood method and 1000 bootstrap replicates based on the general time reversible model [23]. A gamma distribution rate (*g* = 5) was used to model evolutionary differences among sites. A rate variation model allowed some sites to be evolutionary invariable (*I*). Accession numbers are in parenthesis.

**Figure 4:**
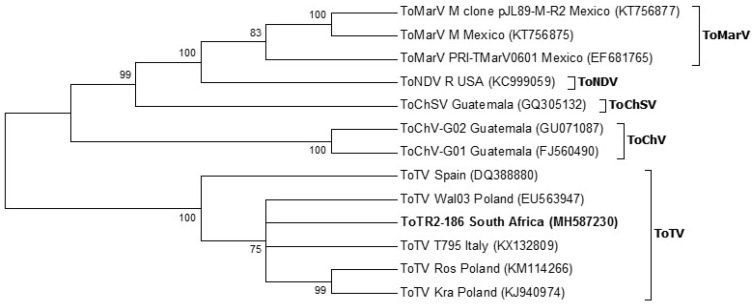
Phylogenetic relationship of the full-length RNA-2 genome from South Africa with all other fully sequenced tomato-infecting torradovirus isolates to date. Evolutionary analysis was carried out in MEGA 6 using the maximum likelihood method and 1000 bootstrap replicates based on the general time reversible model [23]. A gamma distribution rate (*g* = 5) was used to model the evolutionary differences among sites. Accession numbers are in parenthesis.

Phylogenetic analysis of the full-length RNA-1 and RNA-2 nucleotide sequences showed five distinct clades representing the five known members of the torradovirus genus that are capable of infecting tomatoes (Figure 3 and Figure 4); the taxonomy of ToChV, ToChSV, and ToNDV are presently incomplete so these viruses are not approved as distinct species (10th Report of the Internation Committee on Taxonomy of Viruses). The full-length RNA-1 genome of ToTV (ToT-186) from South Africa grouped with ToTV isolates, but it was the most evolutionary diverse. This relationship is supported by strong bootstrap values that are consistent among isolates within the ToTV clade (Figure 3). The tree topology indicates that ToTV may have originated and spread from Poland and Spain to other parts of the world including South Africa.

The full-length RNA-2 genome of ToTV from South Africa (ToT-186) did not cluster with other ToTV isolates. The tree topology in Figure 4 indicates that ToT-186 shares a closer relation to the isolates T795 from Italy and Wal’03 from Poland. The phylogram further outlines the divergence and likely spread of ToTV and other tomato-infecting torradoviruses throughout the world.

Similar tree topologies of the full-length RNA-1 and RNA-2 genomes of tomato-infecting torradoviruses are illustrated in Figure 3 and Figure 4. ToNDV (USA) diverges from ToMarV (Mexico) followed by ToChSV (Guatemala) and finally ToChV (Guatemala). The distance of the branches indicates that ToChV is the most evolutionary diverse species when compared to those mentioned previously. Their distribution is limited to the south-western parts of the United States, Mexico, and Central America. ToTV isolates, on the other hand, form a separate group from all other torradovirus species and have been identified in parts of Europe, Australia, South America, and Africa.

**Figure 5:**
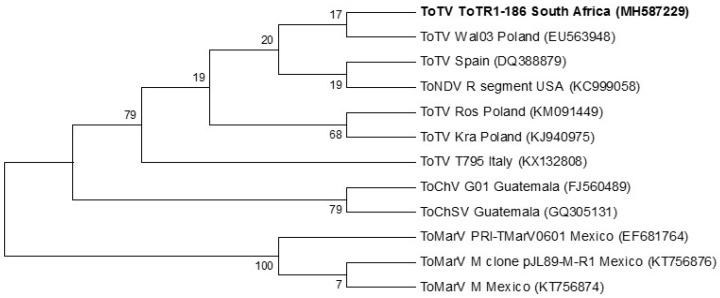
Phylogenetic relationship of the ToTV RNA-1 polyprotein from South Africa (ToTR1-186) in conjunction with all other tomato-infecting torradovirus isolates to date. Evolutionary analysis was inferred using the Maximum likelihood method and 1000 bootstrap replicates based on the General Time Reversible model [23]. Accession numbers are in parenthesis.

Phylogenetic analysis of the torradovirus RNA-1 polyprotein showed that the isolate of ToTV (ToT-186) from South Africa clustered with ToTV Wal03 from Poland; this relationship is supported by a weak bootstrap value (Figure 5). Interestingly, ToNDV groups with ToTV isolates and clusters with the Spanish ToTV isolate (DQ388879). The Italian ToTV isolate T795 diverges from but does not group within the clade of ToTV isolates analyzed in this study. T795 also expressed the highest level of diversity among ToTV isolates based on the tree topology (Figure 5). ToChV and ToChSV clustered and formed their own clade from which a group of ToMarV isolates diverged.

**Figure 6:**
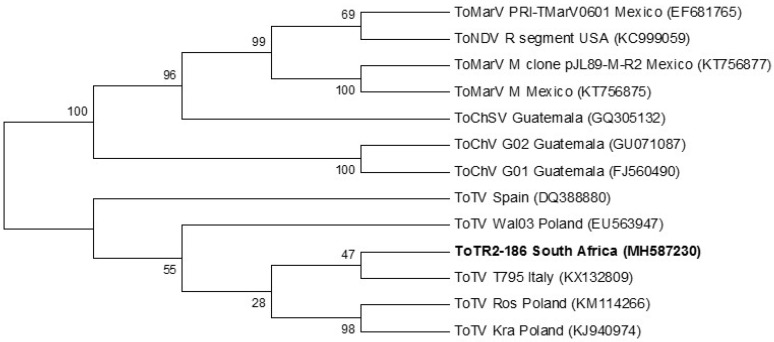
Phylogenetic relationship of the ToTV RNA-2 polyprotein from South Africa in conjunction with all other fully sequenced tomato-infecting torradovirus isolates to date. Evolutionary analysis was carried out in MEGA 6 using the Maximum likelihood method and 1000 bootstrap replicates based on the General Time Reversible model [23]. A gamma distribution rate (*g* = 5) was used to model the evolutionary differences among sites. Accession numbers are in parenthesis.

The tree topology from the phylogenetic analysis of the torradovirus RNA-2 polyprotein shows that ToTV isolates form a distinct clade from other tomato-infecting torradoviruses (Figure 6). The isolate of ToTV (ToT-186) from South Africa clustered with isolate T795 from Italy. The Spanish ToTV isolate (DQ388879) expressed the highest level of diversity and did not group with other ToTV isolates within this clade. ToNDV on the other hand, groups with ToMarV isolates and clusters with the Mexican isolate (PRI-TMarV0601). ToChSV and ToChV isolates diverge from the group of ToMarV isolates, respectively forming separate groups. These relationships are supported by strong bootstrap values (Figure 6).

## 4. Discussion

The recent ToTV outbreak in the northern Limpopo province of South Africa caused substantial damage to fields of commercially grown tomatoes [19]. In the Limpopo province, tomatoes are cultivated on approximately 3600 hectares of farmland which accounts for half of South Africa’s tomato production. Losses to the global tomato industry as a consequence of ToTV infections remain inconclusive. The presence of coinfecting viruses such as Pepino mosaic virus (PepMV) and Tomato chlorosis virus (ToCV) has hampered the efforts of researchers to acquire concise data. The findings of Gomez et al. [24] from their study on ToTV infection of tomato crops in Spain (2005–2008) found that even though most of the crops were singly infected with ToTV, symptom severity was not indifferent to mixed infections with PepMV and other viruses. In addition, they concluded that mixed infections with ToTV and PepMV modulate viral fitness and epidemiology.

In South Africa, tomato crops and weeds that tested positive for ToTV infections were often coinfected with PepMV and ToCV. These co-infecting viruses, i.e., *Potato virus Y* (PVY), PepMV and ToCV were initially identified on samples that were analyzed using high-throughput sequencing. Some tomato crops exhibited burnt-like/necrotic spot symptoms typically associated with torradovirus disease, whilst others displayed interveinal leaf chlorosis and chlorotic flecking symptoms typically associated with crinivirus infections. Symptom expression may be linked to the primary infecting virus and factors that influence viral fitness. Although evidence suggests that there are no significant associations among these viruses (ToTV + PepMV and ToTV + ToCV) [24], the symptomatology of diseased tomato crops masks the presence of coinfecting viruses and facilitates primary spread. Consequently, the dynamics of virus epidemiology are affected, and this may have negative impacts on alternative crop hosts and less tolerant varieties.

Prior to this study, whitefly-transmitted torradovirus disease was unfamiliar to South African farmers and agricultural extension workers. These symptoms were often mistaken for physiological disorders that generally result from the prolonged exposure of crops to higher temperatures or excessive pesticide applications. For many South African farmers, torradovirus infections appeared as an unrelated physiological condition and therefore requires the use of molecular assays to validate symptomatology in the field.

The symptoms associated with ToTV infections are almost indistinguishable from those of other tomato-infecting torradovirus species. Therefore, a generic set of primers [17] was used to screen for the presence of other torradoviruses. Sequence analysis of the 515 bp amplicons indicated that ToTV was the only torradovirus species infecting tomatoes and some weed species in South Africa. High-throughput sequencing of a pooled sample of all ToTV positive nucleic acid extractions confirmed the absence of other torradovirus species in South Africa. Similarly, the absence of whitefly-transmitted viruses was confirmed in symptomatic bell pepper crops infested with whiteflies. These bell peppers did not exhibit typical torradovirus symptoms. Interestingly, the emergence and distribution of ToTV on tomato crops in South Africa was restricted to the Limpopo province.

Sequence analyses of ToTV isolates from the Limpopo region infecting *D*. *stramonium* and tomato crops (Figure 1A–D) were similar, therefore, a sequence was selected and deposited into the NCBI nucleotide database (Lim-186, KP890356) [19]. These results indicate that the epidemiology of ToTV is influenced by the presence of weeds as alternative hosts and a source of virus inoculum. On the other hand, an isolate of ToTV (TorKZN-186, KY581570) identified from samples of the arable weed species (*Abutilon grantii*) growing among tomato field crops in the northern KwaZulu Natal region was not detected on the nearby tomato crops (Figure 1E). This isolate of ToTV may not be a tomato-infecting strain and hence did not pose a threat to tomato production in KwaZulu Natal [9]. Biological assays and host indexing are required to validate the epidemiology of this isolate.

Studies conducted by Alfaro-Fernández et al. [25] reported that ToTV outbreaks in Spain and Poland occurred as a result of high vector populations. During the emergence of ToTV in the Limpopo province of South Africa, whitefly populations reached an unprecedented level. Efforts to control the infestations did not effectively reduce the pest pressure. In addition, South Africa experienced a period of drought with soaring temperatures exceeding 35 °C (95 °F). The warm dry conditions may have influenced the dynamics of whitefly vector populations and fueled their infestation levels. The emergence of ToTV in the warmer and dryer parts of South Africa indicates that climate is an integral part of virus–vector epidemiology. Moreover, South Africa experienced the worst drought in more than 30 years with the highest recorded temperatures in history suggesting that extreme weather events driven by *El Nino* and other climate change phenomena may have contributed toward the emergence of ToTV in the Limpopo province.

ToTV is transmitted by three whitefly species belonging to the genera *Bemisia* and *Trialeurodes*. Both genera were identified in South Africa from adult and nymph phenotypic screening. On the other hand, ToTV can be transmitted mechanically [1], but mechanical inoculation assays on solanaceous hosts including *Solanum lycopersicum*, *Capsicum annuum*, *Nicotiana benthamiana*, *Nicotiana tabacum*, *Nicotiana glutinosa,* and *Solanum melongena* with isolates of ToTV from Limpopo were unsuccessful. Our results indicate that these isolates of ToTV may not be easily mechanically transmissible—a trend generally associated with viruses that are semi-persistently transmitted. Moreover, Pospieszny et al. [26] indicate that ToTV is poorly transmitted mechanically, and this may be attributed to the low stability of ToTV virions in plant sap and low accumulation of the virus in host tissue [27]. Whiteflies collected from the leaf surface of ToTV-positive samples in the Limpopo province constituted a mix of *Bemisia* sp. and *Trialeurodes* sp., but *Bemisia* sp. was more abundant in field samples and to a lesser extent in greenhouses. ToTV-positive weed samples collected from the KwaZulu Natal region were predominantly infested with *Bemisia* sp.

The emergence of ToTV in Australia raised suspicions about the possibility of seed transmission because of the strict import regulations of plant material into the country [28]. Reports of seed transmission of a Polish ToTV isolate at a rate of 0.5% to 0.8% were obtained from seeds of mechanically inoculated pepper and tomato crops [29,30]. Other isolates may show a higher or lower affinity for seed transmissibility, but this is yet to be determined [28]. The exchange of seed and plant material throughout the world is very likely the cause of outbreaks that occur in remote locations from the epicenter of the disease.

The distribution of ToTV in South Africa was restricted to the Limpopo and northern KwaZulu Natal provinces. A significantly higher incidence of ToTV was recorded from the Limpopo province [9]. Although South Africa had a low overall prevalence of ToTV [9], it is important to raise awareness of the symptoms and subsequent economic implications associated with ToTV infections on tomatoes. The distribution of tomato-infecting torradoviruses such as ToMarV, ToChV, and ToChSV has been limited to South and Central America, whereas the occurrence of ToNDV has only been reported from the USA. ToTV is by far the most widespread tomato-infecting torradovirus that has emerged in many European countries, South America, Oceania, and Africa. Recently, another group of torradoviruses known as non-tomato-infecting torradoviruses was identified on hosts such as lettuce, carrot, cassava, and motherwort [28]. The discovery of non-tomato-infecting (NTI) torradoviruses was made possible with the advent of high-throughput sequencing (HTS) technology and raises questions about the genetic diversity, mutation rate, and fitness of torradoviruses. To this end, molecular and evolutionary studies are likely to address these concerns.

The full genome of ToTV infecting tomato crops in the Limpopo province was generated using HTS technology. RNA-1 (ToT-186) from South Africa matched 99% with Spanish (DQ388879) and Polish (Kra (KJ940975)) isolates. Phylogenetic analysis of the full-length RNA-1 genome showed that ToT-186 grouped with other ToTV isolates but was also the most evolutionary diverse (Figure 3). RNA-2 (ToT-186), on the other hand, matched 99% to Italian T795 (KX132809) and Polish Ros (KM114266) isolates. Figure 4 shows that the full-length RNA-2 genome among ToTV isolates are more evolutionary diverse and may be the consequence of coevolutionary events and mutations associated with virulence. In contrast, the phylogenetic relationships among other torradoviruses were more consistent (Figure 3 and Figure 4). The evolutionary distance of each clade indicates that ToMarV is the most distant relative of ToTV. Although only one isolate of ToNDV and ToChSV has been fully sequenced to date, they are likely to be more widespread. The genome of ToNDV was only recently characterized by Wintermantel et al. [6], who claim that it significantly damaged tomato crops in southern California as far back as 1980.

Comparisons of the torradovirus RNA-1 (Figure 5) and RNA-2 (Figure 6) polyproteins were also carried out to account for the disparity associated with varying lengths of different torradovirus isolates and the organizational variation of full-length RNA sequences. Although Figure 5 and Figure 6 display a similar tree topology to that of Figure 3 and Figure 4, respectively, notable variations were identified. ToNDV in particular initially diverged from a group of ToMarV isolates (Figure 3 and Figure 4). Both of these relationships were supported by strong bootstrap values (≥99%); a comparison of the torradovirus RNA-1 polyprotein (Figure 5) showed that ToNDV not only grouped with ToTV isolates but clustered with the Spanish isolate (DQ388879). Interestingly, the RNA-2 polyprotein of ToNDV clustered with the Mexican ToMarV isolate PRI-TMarV0601 within a clade of ToMarV isolates (Figure 6), suggesting that ToNDV may be the result of genetic exchange (recombination/reassortment) between ToTV and ToMarV; no recombination events were detected using recombinant detection program 4 (RDP4) [31].

The representation of the phylogenetic trees in Figure 3 (full-length RNA-1), Figure 4 (full-length RNA-2), Figure 5 (RNA-1 polyprotein), and Figure 6 (RNA-2 polyprotein) indicates that ToTV isolates may have diverged from the other tomato-infecting torradoviruses or vice versa. The branch lengths indicate a significant amount of genetic variation between ToTV isolates and other torradovirus relatives. The results from Table 1 and Table 2 in conjunction with phylogenetic analysis (Figure 3, Figure 4, Figure 5 and Figure 6) demonstrate the evolutionary traits between tomato-infecting ToTV isolates and other torradoviruses analyzed in this study. The movement of ToTV in European and African countries can be inferred from the phylogenetic trees, suggesting that the isolate of ToTV infecting tomato crops in the Limpopo province of South Africa may have originated in Poland and spread from the Mediterranean regions.

Efficient control methods can be designed to contain the spread of ToTV into other provinces of South Africa. Vector control is a key management strategy for emerging viruses such as ToTV which are naturally transmitted by *Bemisia tabaci* and two *Trialeurodes* species. Their coexistence in some locations may be an adaptive or acquired trait or possibly a type of species coevolution that is not entirely suppressing or limiting to the other. It also indicates a level of heightened fitness or the ability to adapt and thrive in the face of competition, extreme weather events, and extensive pesticide use. Weeds need to be managed more effectively, considering their role as alternative hosts. Resistant tomato varieties provide effective control against ToTV infections. These genes can also be introgressed into commercially desirable germplasm via breeding programs. In addition, good crop husbandry, intensive scouting, trap crops, fine mesh nets, mineral oils, pheromones, sticky traps, and pesticides can be used in varying combinations to manage the spread of ToTV. The use of pest phenology models may serve as an early warning system for farmers, especially those who cultivate field-grown vegetables. Importantly, there needs to be a consensus among researchers, government extension workers, farmers, and policymakers so that effective strategies can be implemented based on detailed research outputs and stringent policies that regulate the movement of seed and plant material into South Africa.

## 5. Conclusions

In this study, the prevalence and distribution of ToTV in South Africa are reported. In addition, a comprehensive analysis of the genome of a ToTV isolate from South Africa was generated using high-throughput sequencing technology. The development and use of such technology have led to scientific breakthroughs in many areas of research and may facilitate the identification of many new and emerging diseases. Such was the case of non-tomato-infecting (NTI) torradoviruses. Identification and characterization are the first steps toward developing methods to contain and possibly mitigate diseases such as ToTV. In countries such as Poland and Hungary, ToTV has been totally eradicated.

## Figures and Tables

**Table 1:** A comparison of the nucleotide and amino acid similarities of coding and non-coding regions on the RNA-1 fragment of Tomato torrado virus (ToTV) from South Africa (ToT-186) with all other fully sequenced tomato-infecting torradovirus species identified throughout the world.

Torradovirus Isolate (RNA 1)	5′UTR	Polyprotein		ORF 1 Polyprotein								3′UTR
				Protease co.		Helicase		Protease		RdRP		
	nt (%)	nt (%)	aa (%)	nt (%)	aa (%)	nt (%)	aa (%)	nt (%)	aa (%)	nt (%)	aa (%)	nt (%)
ToT-186 (MH587229) South Africa	100	100	100	100	100	100	100	100	100	100	100	100
ToTV T795 (KX132808) Italy	99.06	99.05	99.72	99.71	100	98.97	100	99.33	100	98.94	100	97.82
ToTV Ros (KM091449) Poland	98.13	99.07	99.30	99.42	99.56	99.31	100	100	100	99.07	100	97.36
ToTV Wal03 (EU563948) Poland	100	98.96	99.53	99.71	100	98.97	100	99.66	100	98.94	99.60	99.50
ToTV Kra (KJ940975) Poland	100	99.10	99.49	99.57	100	98.97	100	99	100	99.07	100	99.67
ToTV (DQ388879) Spain	98.13	98.98	99.58	99.71	100	98.63	100	99.66	100	99.80	100	98.76
ToChV-G01 (FJ560489) Guatemala	72.89	38.20	30.56	N/A	N/A	24.82	14.43	N/A	N/A	72.02	87.95	61.19
ToChSV (GQ305131) Guatemala	71.02	38.01	31.86	N/A	N/A	30.30	21.21	30.34	14.92	24.20	13.09	62.05
ToMarV PRI (EF681764) Mexico	63.55	26.34	13.38	N/A	N/A	79.59	98.96	N/A	N/A	26.23	13.25	N/A
ToMarV pJL89 (KT756876) Mexico	66.35	26.53	13.42	N/A	N/A	79.59	98.96	N/A	N/A	25.97	13.25	61.24
ToMarV M (KT756874) Mexico	66.35	26.56	13.42	N/A	N/A	79.59	98.96	N/A	N/A	26.10	13.25	61.40
ToNDV R (KC999058) USA	66.35	48.56	49.83	N/A	N/A	78.91	97.93	N/A	N/A	N/A	N/A	62.24

nt—nucleotide; aa—amino acid; all values are expressed as a percentage (%); N/A—data not available. ToTV—*Tomato torrado virus*; ToChV—*Tomato chocolàte virus*; ToChSV—*Tomato chocolate spot virus*; ToMarV—*Tomato marchitez virus*; ToNDV—*Tomato necrotic dwarf virus*. Accession numbers for each isolate are listed in parenthesis.

**Table 2:** A comparison of the nucleotide and amino acid similarities of coding and non-coding regions on the RNA-2 fragment of ToTV from South Africa (ToT-186) with all other fully sequenced tomato-infecting torradovirus species identified throughout the world.

Torradovirus Isolate (RNA-2)				ORF-2								
	5′UTR	ORF 1		MP		VP 35		VP 26		VP 23		3′UTR
	nt (%)	nt (%)	aa (%)	nt (%)	aa (%)	nt (%)	aa (%)	nt (%)	aa (%)	nt (%)	aa (%)	nt (%)
ToT-186 (MH587230) South Africa	100	100	100	100	100	100	100	100	100	100	100	100
ToTV T795 (KX132809) Italy	97.34	99.47	98.94	99.08	100	98.78	100	99.43	100	99.38	99.53	99.53
ToTV Ros (KM114266) Poland	97.87	98.95	98.94	98.93	100	98.64	99.18	99.01	99.57	99.53	99.53	99.53
ToTV Wal03 (EU563947) Poland	97.87	99.3	99.47	98.48	100	99.05	100	99.29	100	99.07	100	99.59
ToTV Kra (KJ940974) Poland	97.34	99.47	99.47	98.63	99.08	98.5	99.59	99.15	100	99.53	99.53	99.53
ToTV (DQ388880) Spain	97.34	98.6	98.94	98.93	100	98.91	100	99.01	100	98.92	99.53	99.46
ToChV-G01 (FJ560490) Guatemala	46.27	57.41	70	70.06	80.36	63.27	73.87	73.55	87.28	65.74	81.94	31.43
ToChV-G02 (GU071087) Guatemala	46.27	60.73	71.05	71.27	80.36	63.55	73.87	73.69	86.86	65.59	80.55	31.43
ToChSV (GQ305132) Guatemala	40.42	63.52	73.68	70.36	80.82	64.9	74.69	74.68	91.52	64.36	81.94	49.23
PRI-TMarV0601 (EF681765) Mexico	36.7	60.73	72.1	70.36	80.82	64.22	76.32	73.41	89.83	65.74	79.62	45.88
ToMarV pJL89 (KT756877) Mexico	36.17	60.38	73.68	70.06	81.27	66.66	75.51	73.41	89.4	66.05	80.55	46.62
ToMarV M (KT756875) Mexico	36.17	60.55	73.68	69.9	81.27	66.53	75.51	73.55	89.4	65.89	80.55	46.62
ToNDV R (KC999059) USA	35.63	61.6	70.52	70.06	81.27	64.9	74.28	72.15	91.1	66.35	80.55	46.48

nt—nucleotide; aa—amino acid; all values are expressed as a percentage (%). ToTV—*Tomato torrado virus*; ToChV—*Tomato chocolàte virus*; ToChSV—*Tomato chocolate spot virus*; ToMarV—*Tomato marchitez virus*; ToNRV—*Tomato necrotic dwarf virus*. Accession numbers for each isolate are listed in parenthesis.

## Supplementary Materials

The following are available online at https://www.mdpi.com/1999-4915/12/10/1167/s1, Table S1: Primers used to reconstruct the RNA-1 segment of an isolate of ToTV infecting tomato crops in South Africa., Table S2: Primers used to reconstruct the RNA-2 segment of an isolate of ToTV infecting tomato crops in South Africa.
