# Supplementary material for: Emergence and Full Genome Analysis of Tomato Torrado Virus in South Africa
Source: Viruses. 2020 Oct 15;12(10):1167. doi: 10.3390/v12101167 (PMC7602605; doi:10.3390/v12101167)
Supplement: Supplementary file 1 [file viruses-12-01167-s001.pdf]

Table S1: Primers used to reconstruct the RNA-1 segment of an isolate of ToTV infecting tomato crops in South Africa.

Primer	Sequence (5'-3')	Binding Site (nt)	Tm (°C)	Product size (bp)
ToT1-F1	TAAAAGAGTTATTTTGAGAATATAACC	(1-27)	50	540
ToT1-R1	GAAATTGCAAAAATAAGAAAAGC	(518-540)		
ToT1-F2	GGCTTACTCTTTTGTCGCATCCATG	(379-403)	63	1166
ToT1-R2	CCTCCACTGCTGCCATGGGC	(1525-1544)		
ToT1-F3	GACCGCATGGAAGAGCCAAAGTTG	(1340-1363)	60	822
ToT1-R3	CTCATTACAGCAACCTTGATACAGC	(2137-2161)		
ToT1-F4	GCTTCCCGTGGTTGTTGTAAG	(1942-1962)	59	1001
ToT1-R4	CTTGCTCCTTCAAGAACCGTGC	(2921-2942)		
ToT1-F5	GCAGAAGGAAGAGGAGGGATTACC	(2591-2614)	58	1952
ToT1-R5	GCTTGAGCTGCTATATGTGGTC	(4521-4542)		
ToT1-F6	GTTGGTGGGAGGTATGCCTTC	(4420-4440)	58	1104
ToT1-R6	GCACAACCTCCTGGATATTGAC	(5502-5523)		
ToT1-F7	GCGAACAGCTGCTGCACAGCAG	(5179-5200)	63	1367
ToT1-R7	GCAACACATCTGGGTGTGTTGC	(6524-6545)		
ToT1-F8	GAGCTTTCCAGTGGATCTTGTTGATG	(6271-6296)	60	1233
ToT1-R8	GCACTGTGAAGCCACTGGAC	(7484-7503)		
ToT1-F9	GTTTGAGCGACGACAAAGTT	(7391-7410)	56	97
ToT1-R9	CATATTCAAACTCACACACTGAATTG/Oligo (dT)18	(7762-7787)		

Table S2: Primers used to reconstruct the RNA-2 segment of an isolate of ToTV infecting tomato crops in South Africa.

Primer	Sequence (5'-3')	Binding Site	Tm (°C)	Product size (bp)
ToT2-F1	ATAATTTTATACAATATTTATGTGATCC	(1-28)	50	430
ToT2-R1	CTTAACACCAGTAATTGGCC	(411-430)		
ToT2-F2	GCCCTCAACTGGTTCCTGGTC	(366-386)	60	706
ToT2-R2	CAGGATTGGTTGGATCATTACTGGAAC	(1045-1071)		
ToT2-F3	GCAAACTGGGCATGGGTTC	(814-832)	58	1028
ToT2-R3	CTCCAAGTGGATATTGTGCTTGTC	(1818-1841)		
ToT2-F4	GAGCTGTCGGTGAGACAGCAAGTTG	(1728-1752)	64	633
ToT2-R4	GGAGTTACTGGCCACGCAGGC	(2340-2360)		
ToT2-F5	GCGACAGAGCTGGAATTACAACAAC	(2313-2337)	62	1448
ToT2-R5	GTGATGGCCTCCTGTACCAGTGC	(3738-3760)		
ToT2-F6	GCGACTGTTGGTTTTACCATGCGC	(3686-3709)	62	879
ToT2-R6	CCAGGGAGTGCTCCCCGTAC	(4545-4564)		
ToT2-F7	GATCGTGTACCCTGCTTGGTTAG	(4496-4518)	60	840
ToT2-R7	GCAAAGTTACTGTAGTTCCCGAGTATC	(5309-5335)		
ToT2-F8	GTGTGTTTAATATCATGCATATTTG	(5253-5277)	52	123
ToT2-R8	CATATTCAAACTCACACACTG/Oligo (dT)18	(5355-5375)		
